# Rapid Evolution of Ionic Silver Resistance in *Escherichia* Phage T7

**DOI:** 10.3390/microorganisms14061243

**Published:** 2026-06-01

**Authors:** Larisa Chila Kiki, Monela Ntonifor, Walter LaDelle, Ugonna Morikwe, Franklin Ezeanowai, Lindsey McGee, Akamu Ewunkem, Joseph Graves, Liesl Jeffers-Francis

**Affiliations:** 1 Department of Biology, North Carolina A&T State University, 1601 E Market Street, Greensboro, NC 27411, USA; klchila@aggies.ncat.edu (L.C.K.); mmntonifor@aggies.ncat.edu (M.N.); ucmorikwe@aggies.ncat.edu (U.M.); cfezeanowai@aggies.ncat.edu (F.E.); gravesjl@ncat.edu (J.G.J.); 2 Department of Biotechnology, Alamance Community College, 1247 Jimmie Kerr Rd, Graham, NC 27253, USA; wladelle689@alamancecc.edu; 3 Department of Biology, Biochemistry, Public Health, Quality Science, Earlham College, 801 National Road West, Richmond, IN 47374, USA; mcgeeli@earlham.edu; 4 Department of Biological Sciences, Winston-Salem State University, 601 S Martin Luther King Jr. Drive, Winston Salem, NC 27110, USA; ewunkemaj@wssu.edu

**Keywords:** ionic silver, experimental evolution, T7 bacteriophage

## Abstract

The antimicrobial resistance crisis has led to the use of metals and bacteriophages as possible alternatives to antibiotics. Experimental studies have examined interactions between ionic/nano-silver and bacteriophages against multidrug-resistant bacteria. However, these approaches have often failed to examine whether silver affects the stability and infectivity of bacteriophages. Here, we utilized experimental evolution to evolve resistance to ionic silver in bacteriophage T7. High ionic silver concentrations that do not represent physiological exposure conditions were used to impose strong selective pressure. Evolution of ionic silver resistance in phage T7 was rapid, as evidenced by recovery of bacteriophage growth in *E. coli* following repeated exposures to ionic silver, enhanced infectivity of silver-selected populations relative to parallel control and ancestral populations under increasing ionic silver concentrations, and greater suppression of *E. coli* growth in standard medium. Furthermore, silver resistance evolved without loss of thermal or pH stability under the conditions tested. The genomic foundation of silver resistance was relatively simple, with positive and negative natural selection differentiating the silver-selected populations from the controls and ancestral populations across serial passages in silver. Support for replication-associated adaptation under ionic silver selection may be reflected in recurrent mutations identified in genes involved in transcription, DNA replication, and genome maintenance, including T7p07 (RNA polymerase), T7p10 (DNA ligase), and T7p29 (DNA polymerase I). These findings highlight the importance of evaluating phage –silver combination strategies within an evolutionary framework that accounts for the adaptive capacity of bacteriophages under silver selection.

## 1. Introduction

Ionic silver (Ag^+^) and silver nanoparticles (AgNPs) have been shown to be effective against a variety of multidrug-resistant bacteria in assays lasting between 24 and 48 h [1]. However, the limitation of these studies is that they were not conducted long enough to test the capacity of bacteria to evolve resistance to ionic and nanoparticle silver. A series of simple experiments demonstrated that bacteria can easily evolve resistance to ionic and nanoparticle silver [2,3]. For this reason, similar to other antimicrobial approaches, combination treatments to control multidrug-resistant bacteria have been developed. Combination approaches are most effective when the antimicrobial agents attack aspects of the bacterial phenotype that are not encoded by the same genes [1]. For example, multidrug resistant *Pseudomonas aeruginosa* (*P*. *aeruginosa*) has been effectively treated by a combination of antibiotics and phages [4,5]. Chan et al. [4] used a combination therapy that utilized phage OMKO1 to restore *P. aeruginosa* susceptibility to four classes of antibiotics: ceftazidime, ciprofloxacin, tetracycline, and erythromycin. The primary method of antibiotic resistance in this bacterium was the use of efflux pumps MexAB and MexXY-OprM. However, these are the receptors used by the phage OMKO1 to initiate lytic infection in this bacterium. Chan et al. [4] utilized experimental evolution against phage OMKO1, which resulted in the accumulation of loss of function mutations in the genes encoding the efflux pumps. As a result, the susceptibility to these four classes on antibiotics was restored. Similarly, phage-resistant *Acinetobacter baumannii,* a biofilm-forming bacterium, became susceptible to beta-lactam antibiotics after mutations affecting the phage receptor (the bacterial capsule) impaired capsule production and reduced biofilm formation [6]. Thus, these combination approaches result in an evolutionary trap from which it is difficult for a bacterium to escape. Variants that eliminate the efflux pump or evolve impaired capsule formation result in antibiotic sensitivity, and variants that restore the efflux pump or maintain efficient capsule biosynthesis allow the lytic phage to attack.

Similar approaches are being attempted using both ionic silver (Ag^+^) and AgNPs, although these forms of silver may differ in their interactions with bacteriophages. For example, some researchers are deploying bacteriophages bonded with AgNPs to control biofilms [7,8]. Szymczak et al. [7] evaluated phage T7/AgNP combinations using phage concentrations of approximately 1 × 10^10^ PFU/mL and AgNP concentration of 0.02 mg/mL following a 30 min incubation at room temperature. They demonstrated that phage T7 bonded with AgNPs showed statistically significantly greater reduction of *E. coli* biofilms than controls (no exposure), phage T7 alone, and AgNPs alone after 24 and 48 h of exposure. Similarly, Elsayed et al. [9] showed that phages in combination with AgNPs were more effective at inhibiting the growth of multidrug-resistant *Listeria monocytogenes* (*L*. *monocytogenes*) compared to phages alone or AgNPs alone. Likewise, Lai et al. [10] demonstrated enhanced antimicrobial activity of phage/AgNP antimicrobial coatings against *L. monocytogenes*. Also, some researchers combined AgNPs with engineered temperate phage M13, which displayed a more effective antibacterial activity against *E. coli* compared to AgNP alone or the temperate phage alone with no antibacterial activity [11]. Their work showed the use of phages as vehicles for targeted delivery of AgNP into a specific bacterial host. Independent works by Gilcrease et al. [12] demonstrated that both AgNPs and ionic silver (Ag^+^) can negatively affect phage infectivity. Their study showed that released silver ions reduced phage yields even after nanoparticles were removed from solution, suggesting that ionic silver can impair phage infection dynamics. However, like past studies evaluating the effectiveness of antimicrobials, these studies were conducted using assays that lasted at most 48 h, which may be insufficient to evaluate long-term evolutionary responses. Experimental evolution studies have shown that adaptive mutations emerge progressively across repeated generations under sustained selection pressure. Some mutations arise early and are rapidly lost, whereas beneficial mutations may eventually become fixed within populations [13]. Consequently, short-term assays may capture only the immediate toxic effects of silver without providing a reliable evolutionary assessment of the impact of silver on the capacity of phages to infect their bacterial hosts. This is an important question to answer, as phage–metal combination approaches are becoming popular, and previous studies have shown that exposure to Ag^+^ and AgNPs may impact both viral abilities to infect eukaryotic cells and phage capacity to lyse bacteria [14,15,16,17]. Here, we explored the adaptive evolution of phage T7 to assess potential improvements in its lytic capability against *E. coli* under silver ion pressure. Furthermore, we conducted a genomic analysis to identify the determinants of ionic silver resistance in phage T7.

## 2. Materials and Methods

### 2.1. Bacteriophage and Bacteria

The lytic phage T7 was chosen for this experiment because it is easy to manipulate, has a characteristic short life cycle and can be easily preserved [18]. Bacteriophage T7 (accession NC_001604), kindly provided by Dr. Christina Burch (UNC Chapel Hill), was stored at −80 °C prior to use. An ancestral phage was selected by isolating a single, unique plaque. The genome size for this phage is 39,937 nucleotides. The experimental host was *Escherichia coli* B (ATCC 11303, Manassas, VA, USA), with the genome comprising 4,622,284 base pairs and 4494 annotated genes. An isolated colony was inoculated in 10 mL lysogeny broth (LB) in a 50 mL Erlenmeyer flask and grown overnight in a shaking incubator (MAXQ 400 incubator shaker Thermofisher Scientific, Waltham, MA, USA) set at 37 °C and 150 rpm to an OD600 of 1.1. Aliquots of the culture were resuspended in 50% glycerol (*v*/*v*), stored in the −80 °C freezer and thawed when needed to make active cultures for phage growth.

### 2.2. Plaque Assay

The plaque assay method used in this study was the Double Agar Layer (DAL) method, otherwise called soft agar overlay or double layer method, which is currently the best substantiating test for identifying unique plaque morphology and enumeration [19]. The DAL plaque assay method used in this study was a modification of the protocol described by Kropinski et al. [20]. In our experiment, a one-hundred-fold serial dilution was made by adding 10 µL of phage lysate to 990 µL of LB broth. Diluted phage lysate was then mixed with 300 µL of overnight culture of *E. coli* (OD600 of 1.1) in 3 mL of molten agar maintained at 45 °C (the soft overlay) and poured over a hard agar surface in a petri dish. The petri dish was then incubated overnight at 37 °C. Each plaque represented a single phage infection, and subsequent infection and lysis from its progeny. Plaque morphology (transparent, translucent, halo ring and diameter of clearance) is characteristic of the phage species. While plaques appeared within 3–4 h of incubation, enumeration was performed after 24 h to ensure accurate counting. Plates containing between 0 and 300 plaques were selected, and titers were documented as plaque-forming units per ml (PFU/mL) based on the dilution factor.

### 2.3. Preparation of Ionic Silver (Ag^+^)

Silver nitrate (AgNO_3_) from Honeywell Fluka™ (AgNO_3_; Honeywell Fluka, Charlotte, NC, USA) was the source of Ag^+^ in this experiment. Optimizing the concentration of Ag^+^, the phage exposure time and the incubation time in active *E. coli* medium was necessary to establish the concentration of Ag^+^ to experimentally evolve phage T7. The concentration of Ag^+^ used in this research was 0.1 M, and exposure time was 5 min, during which equal volumes of Ag^+^ solution and phage T7 lysate were mixed. The concentration of Ag^+^ used in this experiment was intentionally high in order to impose a strong population bottleneck on the phage populations.

### 2.4. Experimental Evolution of Ionic Silver Resistance in Bacteriophage T7

**Figure 1:**
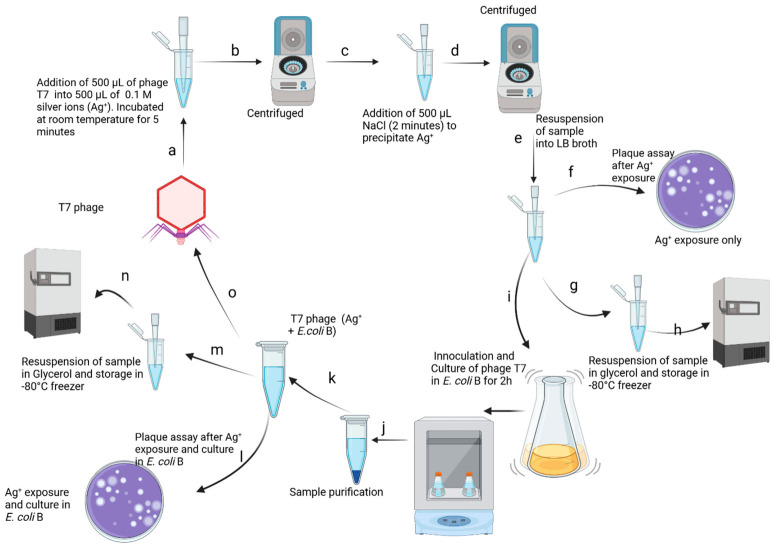
Experimental evolution workflow used to evolve ionic silver resistance in bacteriophage T7. During the ionic silver exposure phase, treatment populations were exposed to 0.1 M Ag^+^ for 5 min at room temperature, while control populations received LB broth instead of Ag^+^ (**a**). Residual Ag^+^ ions were precipitated with 0.1 M NaCl as AgCl following centrifugation steps (**b**–**d**), and surviving phage particles were transferred into LB broth (**e**). Plaque assays were performed to quantify surviving phage populations as PFU/mL (**f**), and aliquots were preserved in 50% glycerol at −80 °C for downstream analyses (**g**,**h**). During the bacterial growth phase, surviving phage populations were inoculated into actively growing *Escherichia coli* B cultures (**i**) and incubated at 37 °C and 150 rpm for 2 h to permit phage replication. Phage lysates were subsequently purified using chloroform treatment and centrifugation (**j**), and resulting populations were transferred into fresh tubes to generate Ag^+^ T7 and Control T7 populations grown in *E. coli* B (**k**). Phage populations recovered after growth in *E. coli* B were used as inocula for the next round of Ag^+^ exposure, completing one serial transfer cycle. A total of 21 serial transfer cycles (G1–G21) were performed during the experimental evolution study. (Created in BioRender.com).

The experimental design for this research was a serial transfer experimental evolution consisting of two phases, the ionic silver exposure phase (Phase 1, also called the metal exposure phase) and the *E. coli* growth phase (Phase 2, also known as reproduction selection phase), as shown in Figure 1.

### 2.5. Phase 1: Ionic Silver Exposure Phase (Metal Exposure Phase)

The lysate from a single plaque of an ancestor strain of phage T7 was divided into 15 independent lineages. Ten lineages were assigned to the treatment group while five parallel lineages served as controls, referred to as T_1_–T_10_ and C_1_–C_5_ respectively. The experimental evolution protocol was conducted on all populations. Before exposure to Ag^+^, the phage titer (concentration) of all lineages was determined by plaque assay. In the treatment group (T), 500 µL of phage lysate was combined with 500 µL of 0.1 M Ag^+^ solution, while in the control group (C), 500 µL of phage lysate was combined with 500 µL of LB broth (1:1 volume ratio) and was incubated at room temperature for 5 min (a). Thereafter, mixtures were centrifuged at 8000 g for 2 min to sediment precipitates (b). Subsequently, 500 µL of the supernatant was transferred into Eppendorf tubes. Into each tube, 500 µL of 0.1 M NaCl was added to bind and precipitate Ag^+^ out of the mixture as AgCl for 2 min (c). These mixtures were centrifuged at 8000 g for 2 min to sediment AgCl (d). Into fresh tubes, 500 µL of the supernatant was then resuspended into 500 µL of LB broth (e). At this point, the ionic silver exposure phase was completed. The first plaque assay was then performed to determine phage concentration as PFU/ mL to be used in the next phase (the *E. coli* growth phase) (f). Plaques from the treatment group were denoted as (Ag^+^ T7), and the controls were denoted (control T7). Stocks of 500 µL were resuspended into 500 µL of 50% glycerol (g) and stored at −80 °C for downstream applications (h).

### 2.6. Phase 2: Growth in Bacteria (Reproduction Selection Phase)

Ten milliliters of LB broth were transferred into flasks, followed by the addition of 300 µL of actively growing *E. coli* stock. Into the treatment and control flasks, 100 µL of phage T7 from the ionic silver exposure step were inoculated accordingly (i). The cultures were then incubated and maintained at 37 °C, 150 rpm for 2 h (approximately 6 generations of phages). After 2 h, 1000 µL of phage lysate from each flask was transferred into a clean Eppendorf tube (j), and 100 µL of chloroform was added to the mixture (to lyse bacteria cells), vortexed and centrifuged at 8000 g for 2 min. The supernatant from the treatment tube was transferred into another tube (G_1_T_1_–G_1_T_10_) to represent Ag^+^T7 grown in *E. coli* (Ag^+^ T7 in *E. coli* B). On the other hand, supernatant from the control tube was transferred into another tube (G_1_C_1_–G_1_C_5_) to represent the control phage T7 grown in *E. coli* B (Control T7 in *E. coli* B) (k). A second plaque assay was performed to determine the phage concentration for the subsequent cycle. The purified stock (500 µL) was mixed with 500 µL of 50% glycerol and stored at −80 °C for future use. This marked the end of the growth in bacteria phase and the end of one complete cycle (G1). A total of twenty-one cycles (G1–G21) were done for this evolutionary exposure experiment.

### 2.7. Silver Tolerance Assay

Two concentrations of Ag^+^ (0.1 M and 0.15 M) and three time points (5, 10 and 20 min) were selected to evaluate silver tolerance in the silver-selected and the control phage populations. Incubation in standard LB medium with no Ag^+^ (0 M) served as the control. For each 21st phage population (treatment and control), 500 µL aliquots were distributed into Eppendorf tubes corresponding to the designated Ag^+^ concentration and incubation time. An equal volume (500 µL) of Ag^+^ was added to each tube, followed by phase 1 processing described previously. After exposure, plaque assays were conducted to quantify phage titers.

### 2.8. Thermal Stability Assay

Three temperature conditions (37 °C, 50 °C and 70 °C) were chosen to evaluate the thermal stability of silver-selected and the control phage populations. Phage populations were maintained at room temperature to serve as controls. For each G21st phage populations, 500 µL aliquots were distributed into five tubes corresponding to the designated temperature treatment. The Fisher thermal heating block was preheated to the desired temperature, and phage lysates were incubated for 30 min and 1 h. Subsequently, plaque assays were performed to quantify phage titers and assess viability.

### 2.9. pH Stability Assay

To evaluate silver-selected phage stability under acidic conditions that mimic the gastrointestinal tract, silver-selected and control phage populations were exposed to LB broth adjusted to pH 3.11 using HCl for two exposure times (30 min and 1 h). Phage populations incubated in LB broth at near-neutral pH (6.68) served as the control for this experiment. For each G21 phage population, 10 µL of phage lysate was added to 990 µL of LB broth at pH 6.68 and 3.11 and incubated for 30 min and 1 h. Then, plaque assays were conducted to measure phage titers.

### 2.10. Phage Lytic Activity Assay

Silver-selected, control and ancestor phage populations were assessed for their bacteria lytic activity. Ten microliters of each phage population (10^8^ PFU/mL) were added to 180 µL of *E. coli* culture (OD600 0.63) in a 96-well plate. An OD600 of 1.0 typically corresponds to approximately 8 × 10^8^ *E. coli* cells/mL; therefore, the bacterial culture used in this assay contained approximately 5 × 10^8^ CFU/mL. This resulted in an estimated multiplicity of infection (MOI) of approximately 0.01, providing an excess of bacterial hosts relative to phage particles and maximizing the likelihood of phage–host interactions. LB broth only was used as a blank, and *E. coli* only was used as the control. The optical density was measured hourly for 24 h using a plate reader (Varioskan ALF multimode microplate reader, Thermo Fischer Scientific, Waltham. MA, USA).

### 2.11. Genomic Analysis

Whole genomic DNA was extracted from the ancestor, treated and control populations of G3, G6, G9, G12 and G21. Extraction was performed using the PureLink^®^ Viral RNA/DNA Mini Kit (Invitrogen, Thermo Fisher Scientific, Waltham, MA, USA) following the manufacturer’s instructions. The purified DNA was quantified using a Promega Quantus fluorometer (Illumina, San Diego, CA, USA), and purity was assessed with a Nanodrop™ spectrophotometer (Thermo Fisher Scientific, Waltham, MA, USA). DNA concentrations in the range of 100–500 ng were used to prepare DNA libraries using the Illumina Nextera XT DNA Library Preparation Kit (Illumina Inc., San Diego, CA, USA), and sequencing was carried out on the Illumina NextSeq™ 2000 platform (Illumina Inc., San Diego, CA, USA). The mean sequencing coverage was ~60,200×, with some outlying samples displaying coverage in excess of 100,000× and few with coverage as low as 200×. Mutations were identified against the phage T7 NC_001604 reference genome using the breseq 0.38.3 pipeline [21]. Whole genome sequencing fastq files are available in the NCBI SRA database using the BioProject ID: PRJNA1441547 and BioSamples: SRR37741538-SRR37741613.

### 2.12. Statistics

Phage concentration was measured in triplicate as PFU/mL, and plaques were manually counted. Plots were constructed using GraphPad Prism software v. 10 (GraphPad Prism 10. Ink). General Linear Analysis (ANOVA) and pairwise *t*-tests were conducted using IBM SPSS v. 25.

## 3. Results

### 3.1. Phenotypic Results

**Figure 2:**
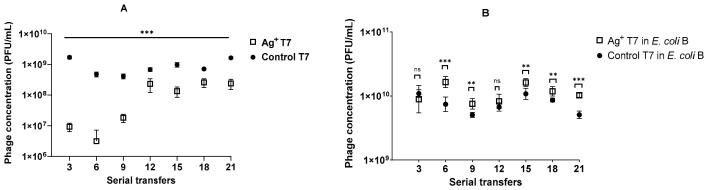
Adaptive responses of phage T7 populations to ionic silver exposure across serial transfers. (**A**) Changes in phage T7 concentrations following exposure to 0.1 M Ag^+^. (**B**) Phage T7 concentration following 2 h growth in *E. coli*. Silver-selected populations (Ag^+^ T7) are represented by open squares, while control populations (Control T7) are represented by black circles. Phage concentrations were determined by plaque assay and expressed as PFU/mL. Data are presented as mean ± SD of biological replicates measured in triplicate. Statistical analysis was performed using two-way ANOVA followed by independent-sample *t*-test for pairwise comparisons, with significance determined at *p* < 0.05. Significance levels are indicated as ** *p* < 0.01, *** *p* < 0.001 and ns (not significant).

Ionic silver demonstrated a significant lytic effect against phage T7, while silver-selected phage T7 populations showed enhanced infectivity toward *E. coli*. Two-way ANOVA revealed significant effects of phage population group (F (1,91) = 2189.88, *p* < 0.001), serial transfer (F (6,91) = 146.09, *p* < 0.001), and their interaction (F (6,91) = 131.06, *p* < 0.001) on phage concentration (Figure 2A). Pairwise comparisons showed significant differences in phage concentration between Ag^+^T7 and Control T7 populations through serial transfers (3–21) (t = −7.96 to −31.46; all *p* < 0.0001) (Figure 2A). Despite the initial decline in phage concentration during early exposures, Ag^+^T7 populations showed progressive recovery across subsequent serial transfers, suggesting adaptive responses of phage T7 to repeated ionic silver exposure. Following 2 h growth in *E. coli*, two-way ANOVA revealed significant effects of phage population group (F (1,91) = 70.34, *p* < 0.001), serial transfer (F (6,91) = 20.08, *p* < 0.001) and their interaction (F (6,91) = 7.67, *p* < 0.001) on phage concentration (Figure 2B). No significant difference in phage concentration was observed between silver-selected and control populations at the 3rd and 12th serial transfers (t (13) = −0.629, *p* = 0.540; and t (13) = 1.70, *p* = 0.113, respectively). However, silver-selected populations produced significantly greater phage concentrations at the 6th, 9th, 15th, 18th, and 21st serial transfers (t (13) = 6.31, *p* < 0.001; t (13) = 3.90, *p* = 0.002; t (13) = 4.48, *p* = 0.001; t (13) = 3.46, *p* = 0.004; and t (13) = 12.84, *p* < 0.001, respectively), suggesting enhanced bacterial infectivity following repeated ionic silver exposure.

**Figure 3:**
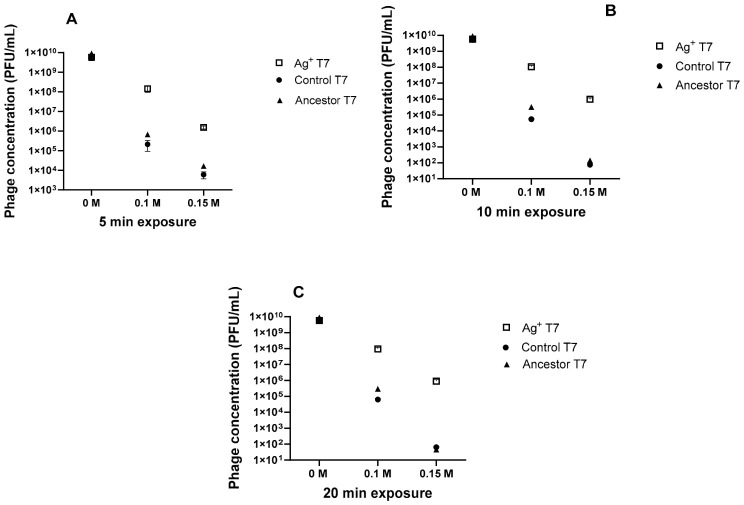
Tolerance of phage T7 populations to increasing Ag^+^ exposure. Phage concentrations were determined before (0 M) and after exposure to Ag^+^ concentrations of 0.1 M and 0.15 M for 5 min (**A**), 10 min (**B**), and 20 min (**C**). Ag^+^ T7 populations are represented by open squares, control populations by black circles, and the ancestral population by black triangles. Phage concentrations were determined by plaque assay and expressed as PFU/mL. Ag^+^ T7 populations consistently maintained higher phage concentrations than control and ancestral populations under increasing Ag^+^ concentrations and exposure times. Data are presented as mean ± SD of biological replicates. Statistical analysis was performed using three-way ANOVA on log10-transformed PFU/mL values, followed by Bonferroni post hoc comparisons, with significance determined at *p* < 0.05.

Silver-selected phages demonstrated enhanced silver tolerance in both a time- and concentration-dependent manner. Figure 3A shows plaque formation for Ag^+^ T7, Control T7, and Ancestor T7 following 5 min exposure to increasing concentrations of Ag^+^. In the absence of Ag^+^, no significant differences in phage concentration were observed among populations. However, at 0.1 M and 0.15 M Ag^+^ concentrations, Ag^+^ T7 populations produced significantly greater plaque concentration compared to control and ancestral populations. Three-way ANOVA performed on log10-transformed PFU/mL values revealed significant effects of phage population (F (2,78) = 5321.00, *p* < 0.001), Ag^+^ concentration (F (1,78) = 2796.81, *p* < 0.001), exposure time (F (2,78) = 168.02, *p* < 0.001), and all interaction effects (all *p* < 0.001) on phage survival. Similar trends were observed following 10 and 20 min exposure periods (Figure 3B,C), where Ag^+^T7 consistently maintained higher phage concentrations under increasing Ag^+^ stress.

**Figure 4:**
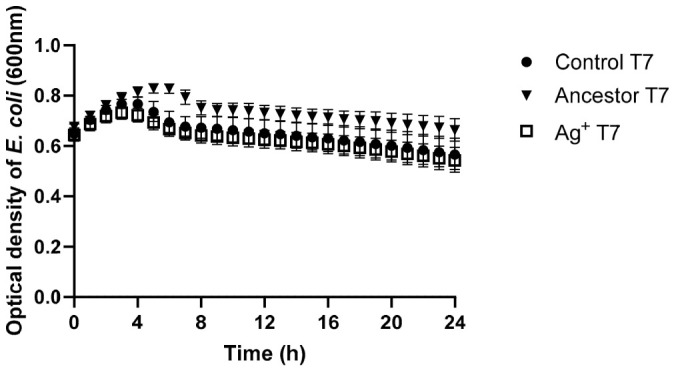
Assessment of phage T7 lytic activity against *E. coli*. Ag^+^T7 (open squares), control populations (black circles), and ancestral phage T7 populations (black triangles) were evaluated for bacterial lytic activity. Optical density (OD600) measurements were recorded at regular time intervals using a microplate reader to assess phage-mediated bacterial lysis. Silver-selected phage populations exhibited enhanced lytic activity against *E. coli* relative to control and ancestral populations. Data represent mean ± SD of 10 treatment biological replicates and five control biological replicates.

Ag^+^ T7 populations exhibited enhanced lytic activity against *E. coli* over the 24 h assay period compared with Control T7 and Ancestor T7 populations (Figure 4). Two-way ANOVA showed significant effects of phage population group (F (2,825) = 579.46, *p* < 0.001), time (F (24,825) = 61.61, *p* < 0.001), and the phage population group × time interaction (F (48,825) = 2.48, *p* < 0.001) on OD600 values. Bonferroni post hoc comparisons showed significant differences among all phage populations (all *p* < 0.001). Ancestor T7 produced the highest OD600 values, followed by Control T7, while Ag^+^ T7 produced the lowest OD600 values, indicating the strongest reduction in bacterial growth.

Thermal stability assays were conducted on ancestor, control, and silver-selected populations, featuring treatments at 37 °C and 50 °C for 30 and 60 min durations. No significant differences in stability were observed across temperature and time. Similarly, no significant differences in stability were observed for pH levels (6.68 and 3.11) for the ancestral, control, or silver-selected phages (Supplementary Figure S1).

### 3.2. Genomic Results

The complete results of the sequencing are provided in Supplemental Tables S2–S6 (Excel format). The ancestor displayed several important variants not encountered in the phage T7 reference genome. This is common as reference genomes are derived from a particular population that was sequenced by an individual researcher or biological supply company [22]. In our ancestor population, nine mutations were fixed (f = 1.000). These included two major deletions: one of 1450 base pairs at position 1257 spanning the genes T7PO1–T7PO6, and a second 75 base pair deletion at position 5849, intergenic between RNA polymerase and a hypothetical protein gene. The remainder of the fixed mutations were single nucleotide polymorphisms (SNPs) (Table S7). Of the nine SNPs that reached fixation, six were non-synonymous, one synonymous, and two were intergenic. There were also six SNPs with frequencies in excess of 0.300, 17 SNPs with frequencies in excess of 0.100, and eight SNPs with frequencies less than 0.100. Within the polymorphic SNPs, 18 were non-synonymous, five synonymous, and five were intergenic. As haploid genomes do not maintain standing genetic variation [22], these variants must have resulted from hard selective sweeps that were in progress in the ancestral environment of the phage T7 sample that was cultured in the Burch laboratory before being utilized in our experiment. For this reason, all genomic results for the controls and silver-selected populations were compared to the genomic variants found in the ancestor population. Theory demonstrates that if these variants make no contribution to phage reproductive success in either the control or silver-selection environment, their frequencies should remain unaltered. However, if they are detrimental to success in the control or silver-selection environment, then their frequency will be reduced by purifying natural selection, and finally should they be beneficial, then we expect their frequency to increase by positive natural selection.

**Figure 5:**
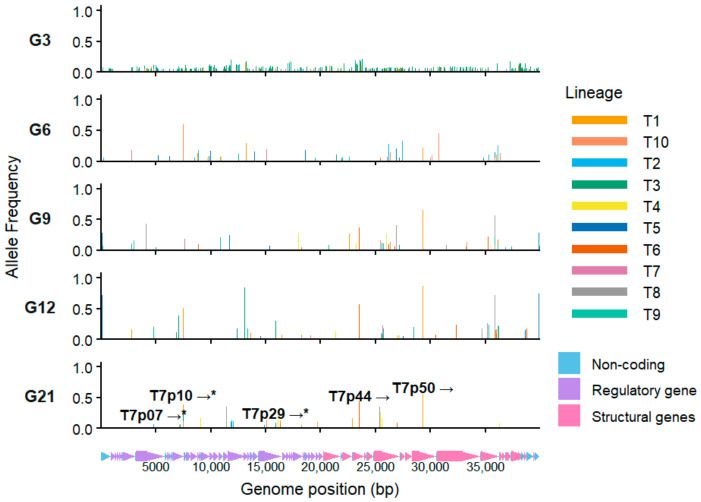
Silver-specific de novo mutations in phage T7 during experimental evolution under silver selection. Allele frequencies of mutations across the phage T7 genome are shown for ten independently evolved lineages (T1–T10) at successive passages for G3 (T5 and T6 excluded), G6 (all lineages included), G9 (all lineages included), G12 (all lineages included), and G21 (T5 and T7 excluded). Each vertical bar represents the allele frequency of a mutation at a given genomic position within a lineage. Mutations are colored by lineage, while the genomic annotation track (bottom panel) indicates non-coding (blue), regulatory (purple), and structural (pink) regions of the phage T7 genome. Only mutations absent in ancestral and control populations are shown. Asterisks (*) indicate parallel mutations occurring independently in multiple lineages. Gene labels with no asterisks (T7p44, and T7p50) highlight recurrent or persistent alleles observed in later passages.

Silver-specific de novo mutations were detected across all serial transfer timepoints, with the highest number observed at G3 followed by a progressive decline across subsequent passages (Figure 5). A total of 288 silver-specific de novo mutations were identified at G3, compared to 41 at G6, 37 at G9, 42 at G12, and 25 at G21. Allele frequency analysis revealed dynamic shifts in mutation frequencies across serial transfers, suggesting ongoing genomic adaptation of phage T7 populations under ionic silver selection. Most of the silver-specific de novo mutations occurred in structural genes (0.505), followed by regulatory genes (0.428), and lastly, non-coding genes (0.067). The mutations in the genes *T7p44* and *T7p50* occurred at earlier timepoints (G9–G21 and G6–G21 respectively) and were maintained throughout the experiment. Genes *T7p07*, *T7p10*, and *T7p29*, marked with asterisks in Figure 5, represent parallel mutations that occurred independently in two or more silver-selected lineages. Table S1 shows the distribution of mutations across genomic regions. At early exposure (G3), the majority of the mutations occurred in the structural genes (0.510), followed by the regulatory genes (0.427), and lastly the non-coding regions (0.062). This trend was similar in G6 (0.487, 0.414 and 0.097), G9 (0.594, 0.297 and 0.108) and G12 (0.595, 0.357 and 0.047). In G21, most of the silver-specific de novo mutations were in the regulatory regions of the genome (0.640), followed by the structural genes (0.320), and lastly the non-coding regions of the genome (0.040).

Control populations retained most ancestral variants during serial transfer G3, with 22 ancestral variants maintained across sequenced control replicates (C_1_, C_3_, C_5_), while 16 ancestral variants were lost in at least one control population. No genomic data was available for replicates C_2_ and C_4_ as their DNA libraries failed to sequence. Only three variants exceeded the frequency of 0.1 (Table S7), and 53 were less than 0.1. There were no major polymorphisms present in the controls at G3 other than those inherited from the ancestor (Table S2).

Silver-selected populations in serial transfer G3 retained 13 ancestral genomic variants and lost 27 ancestral variants in at least one population. No data was available for the replicates T5 and T6 as their DNA libraries failed to sequence. In contrast to the controls, there were 320 de novo mutations in the silver-selected populations, with 59 exceeding a frequency of 0.1 (Table S2). Only variants showing a frequency >0.2 are shown in Table S7 with annotations. Even though silver is thought to interfere with DNA replication, there is no evidence that the presence of silver increases the genome-wide mutation rate in prokaryotes or viruses [23].

Control populations at G21 retained 26 ancestral variants across all replicates, while 14 ancestral variants were lost in at least one replicate. There were 26 variants retained in the control in replicates C_1_, C_3_, C_4_, C_5_ (no data from C2 as its library failed to sequence) (Table S6). The frequency of the ancestral variants remained unchanged for the most part, indicating that these had neutral impacts on viral fitness in the control environment. However, 14 were lost, indicating purifying natural selection acting against them. Table S7 shows the de novo mutations that rose to a frequency > 0.2 at G21. These were not the same variants that arose in G3, indicating that clonal interference replaced the G3 variants at some point during the experiment (see Tables S3–S6).

Silver-selected populations at G21 retained some ancestral variants across all replicates, while additional ancestral variants were lost in at least one replicate population. Of the original ancestral variants, only 16/40 were retained in G21 (Table S6). However, the pattern of ancestral variants lost in G21 was strikingly different from what was observed in G3. In this case, there were 16 variants, which were lost in all silver-selected replicates sequenced (T_1_, T_2_, T_3_, T_4_, T_6_, T_8_, T_9_, T_10_). Most of these were in genes associated with tail fiber formation. This result indicates strong purifying selection against these variants. In addition, there was one variant that was polymorphic in the ancestor in gene *T7p52* that was fixed in all replicates at G21 except for T4 (0.902). This result indicates the opposite (strong positive selection) for this variant. There were 26 de novo mutations in the silver-selected replicates at G21. Those in excess of 0.2 are shown in Table S7 with annotations. The mutations in *T7p22, T7p47*, and *T7p50* first appear in G6 in replicates, T1, T4, and T6, while the mutations in *T7p44* first appeared in G12 (Tables S3–S5).

## 4. Discussion

In this study we have demonstrated that experimental evolution can be utilized to improve the resistance of phage T7 to ionic silver. In G3 (3rd passage), the silver-selected phage replicates displayed a greatly reduced phage concentration upon exposure to ionic silver compared to phages that were not exposed to silver. This exemplifies the anti-bacteriophage property of ionic silver consistent with work done by other researchers [10]. In the works of Lai et al. [10], increasing AgNO_3_ concentrations in paper coatings led to a proportional decrease in *L*. *monocytogenes* phage titers. Our results contrast with You et al. [24], who observed that neither silver nanoparticles nor ions exerted a significant effect on MS2 phage. This could be because our study used an extremely high concentration of ionic silver (0.1 M) to represent the worst-case scenario compared to what was used in their study. However, subsequent exposures demonstrated an increase in the capacity of the silver-selected phage populations to thrive (Figure 2A). In addition, the silver-selected populations rapidly recovered their capacity to replicate in *E. coli* by the 3rd growth phase (G3) and actually performed better than the controls through phases G6–G21 (Figure 2B). Similar to findings by You et al. [24], we observed enhanced phage multiplication, though unlike their study, our system did not involve the addition of silver nanoparticles (AgNPs). While the mechanism by which this occurs is not known, we hypothesize a mechanism similar to that reported by De Plano et al. [11], wherein ionic silver binds on surface phage structural proteins, enhancing the electrostatic attraction between the phage and the host, thereby reducing the adsorption time and hence the replication time. The most powerful demonstration of the silver-selected phages’ resistance to silver was shown by their vastly superior capacity compared to the controls and ancestral phage to lyse bacteria after being challenged with ionic silver (Figure 3). This capacity to lyse bacteria did not come at a cost to phage stability to temperature or pH.

The genomic foundation of ionic silver resistance was demonstrated by evidence of both positive and negative selection. Strong signatures of selective sweeps occurred across the genome of the silver-selected replicates that were not observed in the controls. By G21 the four control replicates had only accumulated four new variants in three genes (*T7p47*, G780R: tail protein, *T7p51*, T892K: internal virion protein, and *T7p52*, G521D: tail fiber protein). These de novo mutations only occurred in two of the control replicates C_1_ and C_4_. In the eight silver-selected replicates there were 20 new variants in 14 genes (and one intergenic). These occurred in seven of the eight replicates (only T_9_ displayed no new variants). There are three possible explanations for the greater number of hard selective sweeps in the silver-selected populations.

First, these variants were discovered because there were twice the number of silver-selected compared to control populations. If the ionic silver did not increase the mutation rate, then we would expect a number of new variants proportional to the number of controls versus silver-selected replicates. However, we observed five times the number, so this disparity cannot be explained simply by the number of variants. The second possibility is that exposure to ionic silver did increase the mutation rate. This is possible because silver is known to interfere with DNA replication [25]. Despite the negative results of silver on the mutation rate reported in Wu et al. [23], there is no reason to believe that this is true in all phages. Third, the mutation rate itself evolves in response to environmental stress. This latter phenomenon is well known from the Long-Term Evolution Experiment (LTEE), in which mutation rate increased in some replicates and impacted the rate of evolution in those lines [13]. An increase in the number of mutations may be beneficial in novel environments [26], and in this study the ionic silver environment represented a novel selective pressure for phage T7. Support for this possibility may be reflected in the mutations observed within genes associated with DNA replication and genome maintenance, including *T7p10* (DNA ligase), *T7p22* (DNA primase/helicase), and *T7p29* (DNA polymerase I) (Figure 5, Table S6). Mutations in these genes may have contributed to replication-associated stress responses or altered mutational accumulation during adaptation to ionic silver exposure.

Negative (purifying) selection also played a role in phage T7 adaptation to silver. By G21 the controls had lost 14 of 40 ancestral variants in at least one replicate. Only the mutations in *T7p42*: position 21132, *T7p50*: position 29806, and *T7p51*: position 34154 were lost in all four control replicates. However, one of these variants originally presented in the ancestor at a frequency = 0.110, and two were at a frequency of <0.100. The latter two could have easily been lost by genetic drift, making negative selection against the variant in *T7p42* (that encodes a tail protein) the most likely candidate for having been acted upon by purifying selection. This result is consistent with the fact that tail fibers are often the target of selection in bacteriophage evolution [27]. On the other hand, the action of purifying selection was much more apparent in the silver-selected replicates. In this case, 24 of 40 ancestral variants were lost in at least one replicate of the eight silver-selected populations. However, 16 of the 24 lost variants were lost in all eight replicates. The fact this occurred in all eight replicates eliminates the possibility that this could have occurred by drift, as the independent probability for such an event approaches zero. Furthermore, only two of the lost variants in the silver-selected populations began at a frequency < 0.100. Finally, of the ancestral variants that were lost in both the controls and the silver-selected populations (*T7p42*: position 21332, *T7p44*: position 23798, *T7p52*: position 35095, *T7p52*: position 35248, *T7p52*: position 35959, and *T7p52*: position 35989), only the *T7p42* variant was lost in all four replicates; whereas, in the silver selected variants, all of these were lost in eight replicates. These findings are consistent with stronger purifying selection acting in the ionic silver environment, as would be expected given the known toxicity of silver.

A limitation of this study is the use of ionic silver concentrations substantially higher than those typically encountered in therapeutic applications. Previous studies evaluating silver-containing wound dressings reported silver release concentrations ranging from approximately 0.0004 ppm to 0.735 ppm in vivo, depending on dressing formulation and application conditions [28]. In contrast, the present study employed 0.1 M Ag^+^ to impose strong selective pressure during experimental evolution. This concentration was intentionally selected to generate a severe population bottleneck and accelerate detectable adaptive responses across serial passages. Consequently, the mutational trajectories and phenotypic responses observed in this study may not fully reflect bacteriophage evolutionary adaptation under physiologically relevant silver exposures.

## 5. Conclusions

There are some clear implications of these results for bioengineering applications concerning ionic and nanoparticle silver along with bacteriophages. First, there is no a priori way of knowing whether combining silver and bacteriophage will reduce/enhance the bacteriophage’s capacity of successfully controlling the targeted pathogenic bacteria. However, given this fact, experimental evolution techniques allow for a rapid method of addressing this potential problem. Phage can be evolved to increase its tolerance for silver, and, as in the case of this experiment, actually improve its efficacy against the target bacterium. With such knowledge, synthetic biology tools can be deployed to more rapidly design phage that can tolerate silver while simultaneously improving their capacity to attack the target pathogens. The use of combination approaches has shown to be a powerful tool against multidrug-resistant bacteria, as the probability of an organism evolving multiple mechanisms of resistance against independent agents of attack (e.g., silver ions and bacteriophages) is much lower than that of either tool alone.

## Data Availability

The whole genome sequencing fastq files presented in this study are available in the NCBI SRA database using the BioProject ID: PRJNA1441547 and BioSamples: SRR37741538-SRR37741613.

## Supplementary Materials

The following supporting information can be downloaded at https://www.mdpi.com/article/10.3390/microorganisms14061243/s1, Figure S1: Thermal and pH stability assay of phage T7 populations after ionic silver exposure title; Table S1: Distribution of mutations across genomic regions; Table S2: Excel sheet of genomic mutations in G3; Table S3: Excel sheet of genomic mutations in G6; Table S4: Excel sheet of genomic mutations in G9; Table S5: Excel sheet of genomic mutations in G12; Table S6: Excel sheet of genomic mutations in G21; Table S7: Excel sheet of genomic annotations.
