# Supplementary material for: Rapid Evolution of Ionic Silver Resistance in Escherichia Phage T7
Source: Microorganisms. 2026 Jun 1;14(6):1243. doi: 10.3390/microorganisms14061243 (PMC13304085; doi:10.3390/microorganisms14061243)
Supplement: Supplementary file 1 [file microorganisms-14-01243-s001.zip › Figure S1.pdf]

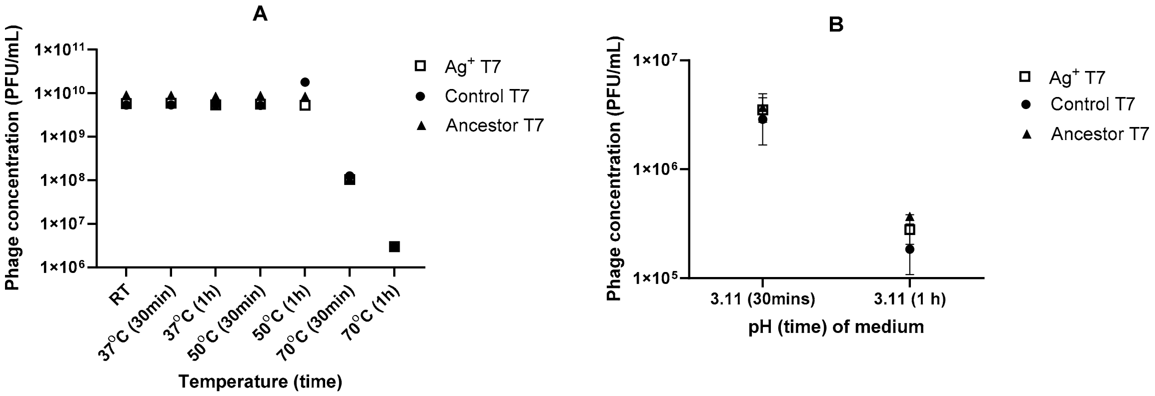

Figure S1. Thermal and pH stability of T7 phage after ionic silver exposure (A) Thermal stability assay showing phage concentrations (PFU/mL) following exposure to room temperature (RT), 37 °C, 50 °C, and 70 °C for 30 min or 1 h. Temperature significantly affected phage concentration $(F(6,91) = 2.37, p = 0.036)$, whereas phage group and the temperature × group interaction were not significant.(B) pH stability assay showing phage concentrations following exposure to neutral pH (6.68) and acidic pH (3.11) for 30 min or 1 h. pH condition significantly affected PFU concentration $(F(1,26) = 50.47, p < 0.001)$, whereas phage group and the pH × group interaction were not significant. These findings indicate that acidic conditions substantially reduce phage infectivity across all populations under the tested conditions. Data are presented as mean ± SD. Open squares represent silver-selected T7 phages, closed circles represent control T7 phages, and closed triangles represent ancestor T7 phages.
