# Supplementary material for: Rapid Evolution of Ionic Silver Resistance in Escherichia Phage T7
Source: Microorganisms. 2026 Jun 1;14(6):1243. doi: 10.3390/microorganisms14061243 (PMC13304085; doi:10.3390/microorganisms14061243)
Supplement: Supplementary file 1 [file microorganisms-14-01243-s001.zip › Table S1.pdf]

Rapid Evolution of Ionic Silver Resistance in *Escherichia* phage T7

Table S1: Distribution of de novo mutations across functional genomic regions of T7 bacteriophage under silver selection (G3–G21).

Passage	Gene Category	Number of mutations	Percentage (%)
**G3**	Non-coding	18	6.25
	Regulatory	123	42.71
	Structural	147	51.04
**G6**	Non-coding	4	9.76
	Regulatory	17	41.46
	Structural	20	48.78
**G9**	Non-coding	4	10.81
	Regulatory	11	29.73
	Structural	22	59.46
**G12**	Non-coding	2	4.76
	Regulatory	15	35.71
	Structural	25	59.53
**G21**	Non-coding	1	4.00
	Regulatory	16	64.00
	Structural	8	32.00
